# The Combined Use of Liquid Fertilizer and Urease/Nitrification Inhibitors on Maize Yield, Nitrogen Loss and Utilization in the Mollisol Region

**DOI:** 10.3390/plants12071486

**Published:** 2023-03-28

**Authors:** Xiaoyu Li, Xiaoyan Zhang, Shaojie Wang, Wenfeng Hou, Li Yan

**Affiliations:** Key Laboratory of Straw Comprehensive Utilization and Black Soil Conservation, College of Resources and Environmental Sciences, Jilin Agricultural University, Changchun 130118, China

**Keywords:** UAN, N_2_O and NH_3_ losses, urease and nitrification inhibitors, N recovery efficiency, N budgets

## Abstract

Nitrification inhibitor (NI) and urease inhibitor (UI) with fertilizer have the potential to reduce nitrogen (N) loss as well as improve grain yields. Urea–ammonium nitrate (UAN) solution as liquid fertilizer is superior to conventional solid nitrogen (N) fertilizer in terms of fertilizer efficiency, energy savings, environmental pollution reduction and economic benefits. However, comprehensive assessments of UAN with inhibitors from an environmental and agronomy perspective, including insights into the mechanisms of UAN with inhibitors, are lacking. In a field trial, three single-inhibitor and two double-inhibitor (DI) treatments were set to quantify the grain yield, the N losses and the N recovery efficiency of maize treated with urea supplemented with dicyandiamide (DCD), 3,4-dimethylpyrazole phosphate (DMPP) and N-(n-buty_l_) thiophosphoric triamide (NBPT). Compared with the UAN treatment, the supply of urease inhibitors reduced NH_3_ emission by 13.0% but increased N_2_O emission by 13.0%. The supply of nitrification inhibitors delayed the conversion of ammonium N to nitrate N and improved NH_3_ emission by 23.5–28.7%, but reduced N_2_O emission by 31.4% and significantly increased the maize yield by 21.3%. The combined use of NBPT and DCD were not compatible in UAN and cannot achieve the maximum potential for optimizing yields and reducing nitrogen losses. Considering the grain yield, the N use efficiency and the N losses, the combined use of NBPT and DMPP in maize production system significantly improved the grain yield and N use efficiency, as well as reduced N losses.

## 1. Introduction

Improving nitrogen (N) management to increase agricultural production while minimizing unintended environmental consequences is critical for feeding the growing population [1]. Urea is by far the predominant N fertilizer used worldwide [2]. Depending on fertilizer managements, environmental conditions (temperature, wind speed, rainfall, etc.) and soil properties (calcium content, cation exchange capacity, acidity, etc.), N loss due to NH_3_ emissions can range from 0–50% [3,4]. In fact, N loss through NH_3_ emissions is a very important environmental component in Life Cycle Assessment (LCA) of agricultural systems. Brentrup et al. (2001) compared the bio-economy of two different N fertilizers (urea–ammonium nitrate (UAN) and urea) on sugar beet using the LCA methodology; it was indicated that the value of the UAN eco-indicator was significantly lower than that of urea [5]. UAN is made up of urea, ammonium nitrate and water, which is abided with three N forms and has the advantages of being a quick and long-acting N source and producing an enhanced N utilization rate [6]. Research in North America has shown that the UAN has significant effects on crops development [7]. The yield of maize was increased by 8.4%, the N uptake was increased by 13.3% and the N residue in the soil was reduced by 26.6% for the supply of UAN [8,9,10,11,12,13]. Spalding et al. (2019) also found that the nitrate contamination in 38 wells was mostly from nitrified ammonium fertilizer [14]. Most importantly, nitrate fertilizer from UAN was not isotopically identified in groundwater beneath nearly all fields with documented heavy UAN use, which could be a potentially valuable finding for N fertilizer managements. However, UAN has also exhibited a high NH_3_ emissions (9.5–12.6%) after field application [15], which calls for mitigation measures.

In light of the limited N recovery efficiency of UAN, there has been increased interest in using urease inhibitor (UI) and nitrification inhibitors (NI). These include slow-release fertilizers such as polymer-coated urea and N fertilizers with chemicals to inhibit biological processes such as hydrolysis of urea and oxidation of NH_4_^+^ [16]. Several measures have been developed to reduce the N emissions, and the application of UI to slow down urea hydrolysis is one of the most robust [17]. UI temporarily inhibited urease and controlled the release of N in the soil-water matrix, allowing a better synchrony between N supply and crop demand, which ultimately improves the N recovery efficiency (NRE) and minimizes gaseous loss from N fertilizer [18,19]. In the 1980s, nearly 70 kinds of UI were developed internationally [20]. The inhibitory effect of hydroquinone in dihydric phenols and terpenoids in organic compounds is the most ideal, and N-(n-butyl) thiophosphoric triamide (NBPT) in phosphoramides also works better [21]. It was reported that the supply of UI to UAN reduced the urea hydrolysis and emissions of ammonia [22]. In summary, studies have shown that the combining UI with UAN could effectively reduce the ammonia emissions. NIs are organic or inorganic compounds that inhibit ammonia-oxidizing bacteria activity and thus inhibit the oxidation of NH_3_ to nitrite [23]. Amberger (2008) pointed out the roles of NI in soil and concluded that the application of NI could both increase the NRE and reduce the N emission to the environment. The United States took the lead in carrying out the artificial synthesis of NI, but it was promoted as an agricultural material until the 1980s [24]. McCarty and Bremner (1989) found that alkanes, alkenes, alkynes, aromatic hydrocarbons and the derivatives of four N heterocyclic compounds also have the property of inhibiting nitrification [25]. Subsequently, Japan and Germany and other countries have conducted in-depth research on NI and have developed thiourea (TU), 3,4-dimethylpyrazole phosphate (DMPP) and other products that are widely used in agricultural production practices in Europe, South America, Australia, North Africa and Asia [26]. At present, NI with patents and registered trademarks circulated in the market are very rich, but only Nitrapyrin (chloromethyl pyridine), dicyandiamide (DCD) and DMPP are widely used in agriculture production systems. Lam et al. (2017) also discussed whether the addition of NI to urea and organic fertilizers can increase NH_3_ emissions due to a prolonged time with high concentrations of NH_4_^+^ in the soil [27]. To avoid the negative effects, the combination of NI and UI in urea is an option to control the loss pathways. The positive effects of UAN on both crop production and reduction in NH_3_ and N_2_O emissions have been reported [28]. There are also N products that combine a urease and nitrification inhibitor (UI + NI) added to UAN [1,29]. The goal of these dual inhibitors is to inhibit both urea hydrolysis and nitrification and then reduce the NH_3_ emission, nitrate N leaching and ultimately keep the N in the soil of rooting zone as long as possible [30]. Previous studies have shown that the combined use of UI and NI in UAN significantly decreased the NH_3_ and N_2_O emission [31].

In this study, field trials were designed in the Mollisol region in China for two consecutive years to study the interactive effects of different inhibitors in UAN on maize yield, plant N uptake, soil inorganic N residue and N balance. The objectives of this study were to (1) monitor the N_2_O and NH_3_ emissions for the combination of UAN with UI and/or NI; (2) identify the effects of the combination of UAN with UI and/or NI on maize yield, N uptake and N efficiency; (3) evaluate the residual N (NO_3_^−^ and NH_4_^+^) in soil.

## 2. Results

### 2.1. Grain Yield

**Figure 1:**
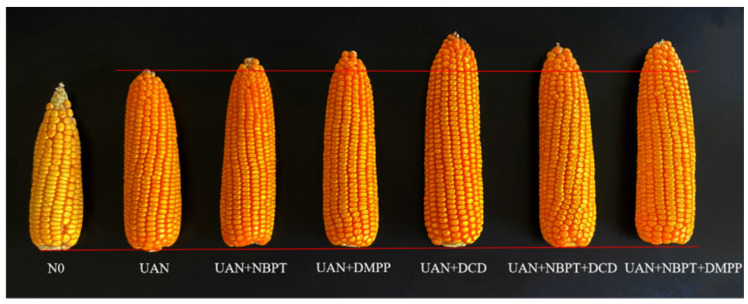
Morphological effect of UAN and inhibitors on maize ear.

**Figure 2:**
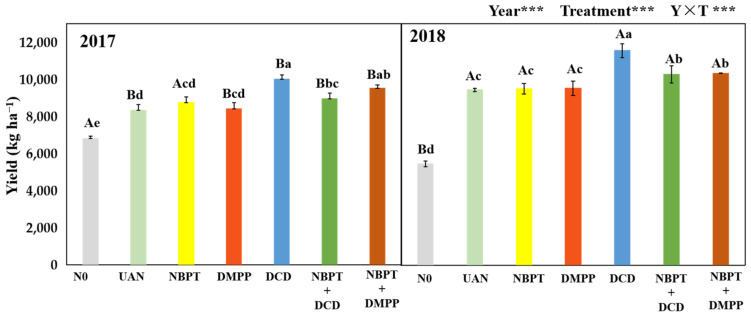
Maize yield of the different sources of N fertilizer in 2017 and 2018. Note: *** indicate significance at *p* < 0.001. Different capital letters above the bars indicate significant differences among years at *p* < 0.05. Different lowercase letters above the bars indicate significant differences among treatments at *p* < 0.05. Letters on the top of the histogram marked with at least the same letter do not differ significantly (*p* = 0.05).

The supply of N significantly increased the maize yield (Figure 1). Compared with the N0 treatment, the supply of N significantly increased the maize yield by 32.3–85.2% across two years (Figure 2). Compared with the UAN, UAN supplemented with NBPT or DMPP had no significant effects on grain yield. Conversely, UAN supplemented with DCD had significant effects on grain yield, which was increased by 20.1% in 2017 and 22.4% in 2018, respectively. Similarly to NBPT + DCD and NBPT + DMPP was significantly increased by 7.4% and 14.3% in 2017, 8.8% and 9.5% in 2018, respectively. Interestingly, the NBPT + DCD showed relative lower yield increase rate than that of DCD.

### 2.2. NH_3_ and N_2_O

**Figure 3:**
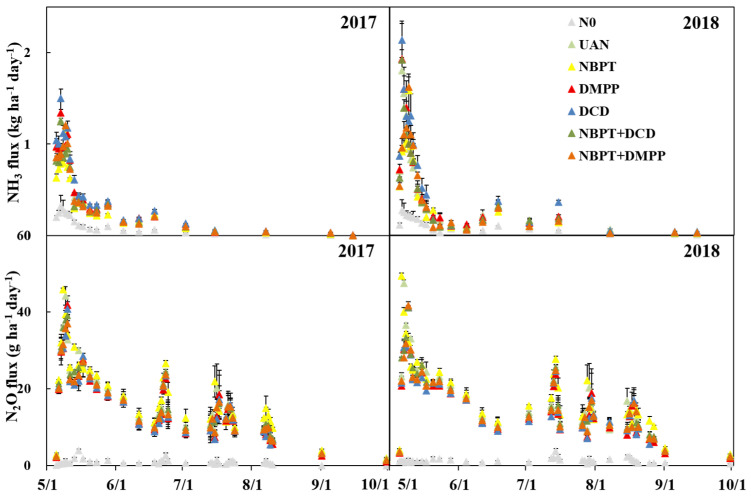
NH_3_ flux and N_2_O after fertilization, bars are standard error of the mean.

The dynamic fluxes of NH_3_ and N_2_O emission were shown in Figure 3. The rate of NH_3_ flux peaked at 2–5 days after fertilization and then dropped sharply within the next weeks, but the peak period of NBPT and NBPT + DMPP was postponed about 2–3 days. The mean NH_3_ flux of the UAN was 1.6 kg ha^−1^ d^−1^; while only 1.2 and 1.5 kg ha^−1^ d^−1^ in the UI and 2 double-inhibitor, 1.7 kg ha^−1^ d^−1^ was observed in the NI. The mean NH_3_ flux rates of the UAN across two years was 0.47 kg ha^−1^ d^−1^, which was significantly higher than the 0.40, 0.46 and 0.45 kg ha^−1^ d^−1^ of the NBPT, NBPT + DCD and NBPT + DMPP treatments, respectively, and significantly lower than the 0.51 and 0.57 kg ha^−1^ d^−1^ of the DMPP and DCD treatments. The rate of N_2_O flux showed four significant peaks during the growth period. The rate of N_2_O flux peaked at 4–6 days after fertilization at first and then dropped sharply within the next weeks in 2017, but the peak advanced two days due to the rainfall in 2018. The peak of NBPT was advanced one day than UAN, and other treatments was postponed one day than UAN. The mean N_2_O flux rates of the UAN were 191.3 kg ha^−1^ d^−1^, while only 198.5 kg ha^−1^ d^−1^ was observed in the UI; 172.1 kg ha^−1^ d^−1^ and 165.5 kg ha^−1^ d^−1^ were observed in the DI and NI.

The total emission of NH_3_ and N_2_O were shown in Table 1. The total NH_3_ emissions of N-fertilized treatments were 16.2–22.6 and 18.5–27.8 kg N ha^−1^ in 2017 and 2018, respectively, accounting for 6.1–9.7% and 6.9–12.1% of the total N application rates. The amount of NH_3_ emission varied remarkably with different inhibitors. Compared with the UAN treatment, addition with NBPT significantly reduced the NH_3_ emission by 11.5% in 2017 and 14.4% in 2018, respectively, while DCD and DMPP significantly increased the NH_3_ emission losses by 10.9% and 23.5% in 2017, and 7.4% and 28.7% in 2018, respectively. The total N_2_O emission of N-fertilized treatments were 1.51–1.91 and 1.71–2.08 kg N ha^−1^ in 2017 and 2018, respectively, accounting for 0.81–1.01% and 0.85–1.06% of the total N application rates. Compared with the UAN treatment, the NI and DI treatments significantly reduced N_2_O emissions by 26.9–35.8% in 2017 and 12.0–17.5% in 2018, respectively. Compared with the UI, the NH_3_ emission of NI was significantly increased by 35.3%, while the N_2_O emission was decreased by 18.7%. Compared with the UAN, the NBPT + DMPP significantly reduced the NH_3_ and N_2_O emission by 7.5% and 7.4%, respectively.

### 2.3. Soil NH_4_^+^-N and NO_3_^−^-N

**Figure 4:**
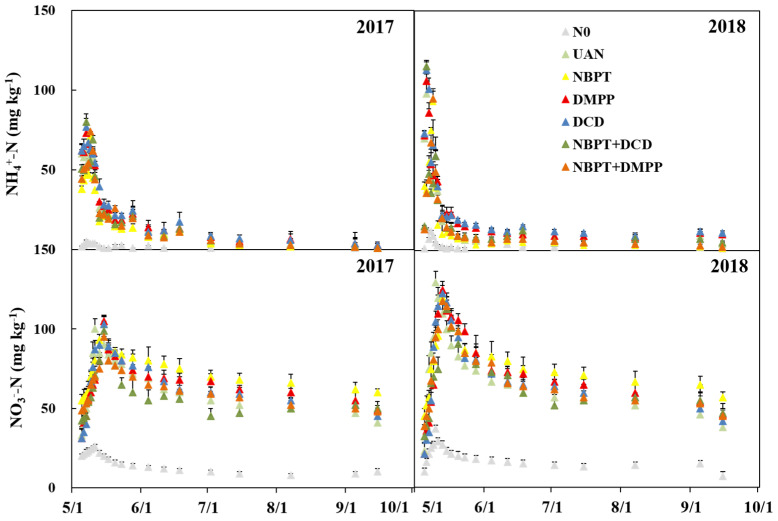
NH_4_^+^-N and NO_3_^−^-N content in the soil of different fertilizer after fertilization, bars are standard error of the mean.

NH_4_^+^-N and NO_3_^−^-N contents in the top 20 cm in the soil showed a high response to N fertilizer (Figure 4). Compared with the UAN, NH_4_^+^-N contents in the soil of DMPP and DCD increased by 3.2% and 5.5% in 2017, and 4.8% and 8.3% in 2018, respectively. In contrast, NH_4_^+^-N contents in the soil of NBPT, NBPT + DCD and NBPT + DMPP decreased by 18.4%, 5.6% and 6.6% in 2017, and 20.9%, 9.2% and 10.8% in 2018, respectively. Compared with the UAN, the soil NO_3_^−^-N contents of 3.6% was reduced by DI, and that of UI was increased by 13.8% in 2017, and 8.8% in 2018, respectively.

### 2.4. Distributions of Residual N in Soil

**Figure 5:**
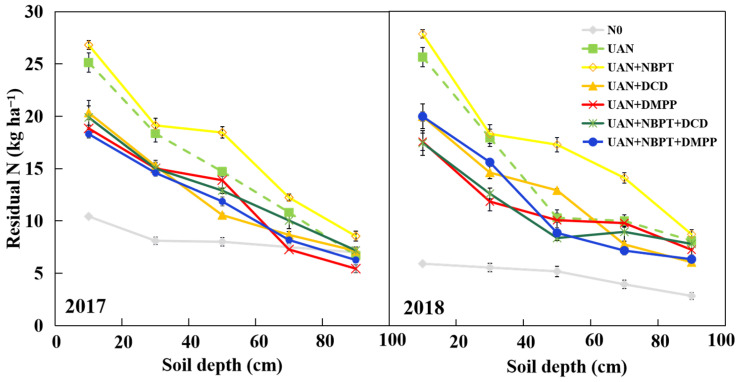
Distributions of soil residual nitrogen in the 0–100 cm soil; bars are standard error of the mean.

On the whole, there were 59–76 kg ha^−1^ inorganic N remaining in the 0–100 cm soil layer after maize harvest in 2017 and 55–72 kg ha^−1^ in 2018 (Table 2). Compared with the UAN, NBPT significantly increased residual N by 11.8% and 19.4% in two years, respectively, and other inhibitors addition decreased residual N in the 0–100 cm soil layer by 16.6–20.9% across two years. The residual N in soil mainly remained at the 0–40 cm depth, accounting for 53.7–57.4% of the total residual amount in 2017, and 52.0–61.4% in 2018 (Figure 5). In addition, the amount of residual N in the middle and deeper soil layers (40–100 cm) of N treatments except for NBPT were close to N0 in 2017. However, all N treatments were significantly higher than N0 except in the 80–100 cm soil layer in 2018. The results showed that NBPT could increase N leaching risk due to the higher residual amount in soil, NI and DI could effectively prevent fertilizer N leaching, increase the residual amount in the upper soil layer and in turn increase the uptake opportunity by crops.

### 2.5. Nitrogen Budget in a Soil-Maize System

The total N uptake varied from 181.5 to 205.5 kg ha^−1^ in the fertilized treatments, and inhibitor application significantly increased the total N uptake (Table 2). NBPT + DMPP treatment showed the highest N uptake, which was 203 and 208 kg ha^−1^ in 2017 and 2018, respectively. The N budgets were calculated in Table 2. In 2017 and 2018, the average inorganic N amounts in the 0–100 cm soil layer before the sowing were 93 and 64 kg ha^−1^, respectively. The apparent N losses of DCD were approximately 12–27 kg ha^−1^, lower than that of UAN. The residual N in 2017 (averaging 85 kg ha^−1^) were all higher than those in 2018 (averaging 45 kg ha^−1^). The “apparent N losses” in Table 2 comprise all losses except NH_3_ and N_2_O losses, including N leaching and/or losses due to nitrification/denitrification, as well as possible errors.

The nitrogen recovery efficiency (NRE) of DCD was the highest across two years, which was significantly higher than that of DMPP and NBPT, and the UAN was the lowest (Table 3). The apparent residual N was the lowest with NBPT + DMPP, followed by DMPP and NBPT + DCD, while NBPT was relatively high. The apparent N loss rate of UAN and DMPP was higher. The apparent N utilization rates of DCD and NBPT + DMPP were higher than other treatments, while the apparent residual N was relatively low. The results showed that the DCD and NBPT + DMPP had less N residue and losses with higher NRE. The average NRE of UI, NI and DI treatments was 38.1%, 45.4% and 46.6%, respectively, across two years.

## 3. Discussion

Our study indicated that ability to increase the yield and reduce the emissions of the liquid fertilizers and different types of inhibitors varied in the maize production system of northeastern China.

### 3.1. Grain Yields of UAN Combined Inhibitors

Previous studies have shown different types of combined UAN inhibitors can increase the grain yields [32]; this is not exactly the same as the result of this study. The results of UAN combined with UI showed maize grain yields reduced; similar results also were reported by Woodley et al. (2018) [33]. This was mainly because the UI slowed down the hydrolysis of the urea and reduced the concentration of NH_4_^+^, thus limiting the rate of nitrification and the supply of NO_3_^−^. At present, there are few studies on UAN combined with NI; most studies focused on solid fertilizer combined with NIs [29]. Our results showed that the yield increase rate of UAN combined with DCD was 8.2% higher than that of DMPP, indicating that DCD was more suitable for UAN when adding a single nitrification inhibitor. In addition, our results showed that, compared with UAN, DI treatments (NBPT + DCD, NBPT + DMPP) significantly increased maize grain yields by 8.1–14.1%; in contrast, Drury et al. (2017) found a 7% increase in maize grain yields when using UI + NI with UAN, compared to UAN alone; the reason for the difference is the increase in N loss caused by soil type [34]. 

### 3.2. NH_3_ and N_2_O Emission of UAN Combined Inhibitors

In the present study, the NH_3_ emission factor in the UAN accounted for 8.0%; these results are similar to those of the previous studies [17]. NH_3_ emission of UAN and UAN + NBPT was 11.1% and 9.5% of the N application rate, respectively, which was significantly lower than that reported in the previous study [33]. Silva et al. (2017) revealed an accumulated NH_3_ emission of urea and UAN + NBPT could reach 31% and 15% of the N rate, respectively, in a wide range of soil, weather and management conditions [25]. These results confirmed that NH_3_ emission has a substantial loss of N from agricultural systems, which could be mitigated by adopting appropriate fertilizer products and/or optimized management practices. Similar to those of a large number of solid fertilizer experiments, our results showed that the addition of a UI to liquid fertilizer can effectively inhibitt the NH_3_ emission, but adding an NI increased the NH_3_ emission [34]. In a short time, the single application of UI only delayed the time of the formation of volatile ammonia, but cannot reduce the total ammonia emission loss [35,36,37]. Only by combining two types of inhibitors and exerting their synergistic effects can the whole process of urea N conversion in the soil be effectively adjusted, thereby reducing the loss of urea N by various pathways.

At present, research on the mixed application of DI is abundant. There are also dual-inhibitor products such as Agrotain Plus and Super U on the market, both of which are a mixture of the NBPT and the DCD. However, the effects of the combined application of the two inhibitors are still unclear [26,29]. Some studies have reported that the simultaneous application of the two inhibitors not only reduces NH_3_ emission, but also reduces soil nitrate accumulation and N_2_O emission [31,38]. In this research, DI could reduce the NH_3_ and N_2_O emission by 3% and 6% compared with the UAN, respectively. Although there are also reports that mixed applications of both could stimulate the breakdown of NI and increases the risk of nitrate leaching [39,40]. Li et al. (2018) indicated that there may be an antagonistic effect between the two inhibitors; especially in dry soils with higher pH, the mixed application did not produce a synergistic effect [1]. In addition, the suitable climate and soil conditions for the two inhibitors are different. NI effectively reduces N loss in low-temperature areas and acidic soils, while the UI is more suitable for application in areas with higher annual average temperatures and moderately alkaline soils. Therefore, the suitable conditions are inconsistent between the two inhibitors.

### 3.3. N Losses of UAN Combined Inhibitors

In addition to the large portion of apparent N losses due to NH_3_ and N_2_O emission, the large amounts of N accounted for other loss pathways, such as N leaching and nitrification/denitrification [30]. UI and UI + NI mainly reduce NH_3_ emission, and there is no evidence for the effects on decreasing other N losses pathways. Apparent N losses were the greatest for UAN and UAN + DMPP in this study, which indicated that the highest N losses and N surpluses in them. The N budgets of UAN + DMPP indicated that 62 kg N ha^−1^ residual nitrate N in 2017 was retained in the soil after harvest, by up to one third of the applied N rate. Part of residual soil nitrate N in the soil may be subject to environmental loss, particularly in heavy rainfall or flood irrigation period, prior to the rapid growth stage of the summer maize. A meta-analysis of the application of NI in different regions found that the application of NI significantly reduced the N_2_O and NO emission by 44% and 24%, respectively, reduced nitrate leaching losses by 48%, and increased NH_3_ emission by 20%; in total, NI reduced the total N content by 16.5% while significantly increasing economic benefits [41]. The NI studied were further subdivided to focus on a meta-analysis of the current mainstream NI, DCD and DMPP [42]. The application of NI can effectively reduce the greenhouse gas emissions, reduce the N losses and improve the economic efficiency. Therefore, manufacturers and R&D personnel try to mix the two inhibitors to achieve a decrease in multiple losses and synergistic emission reductions. However, in the case of DI, one plus one was not more than two. According to the comprehensive analysis of the effects of multisite field experiments, the DI were not as effective as single inhibitors in preventing NH_3_ and NO_3_ leaching, and some studies found that all N had leached from the soils [9]. Combined use of NBPT and DCD on alkaline soils increase nitrate leaching because the different environmental conditions suitable for application and chemical reactions failed [39,40,43,44]. Therefore, it may be a better solution to combine UI and NI based on the characteristics of N loss at different crop growth stages. Synergistic N fertilizer is the direction in which the N fertilizer industry is moving, but its application effect is greatly affected by soil and climatic conditions. At present, the best application method is relatively uncertain, and field verification is needed to guide the application of synergistic N fertilizers.

The validation of the application of synergistic N fertilizers in the field has been in progress for nearly 40 years [20]. Current studies are spread all over the world and include a variety of crop systems [37]. However, the application of synergistic N fertilizer has been reported to have both positive and negative effects in many studies. In some areas and crops, the application of N fertilizer has been shown to have a significant increase in production and synergy, but there is no obvious gain effect in other crop systems or soil environments [1,17]. This variability also leads to surrounding comprehensive benefits ambiguity of synergistic N fertilizers. In summary, the application of UAN combined with inhibitor can not only reduce the N losses and improve the ecological benefits, but also increase the yield production and improve the economic efficiency.

## 4. Materials and Methods

### 4.1. Site Description

**Figure 6:**
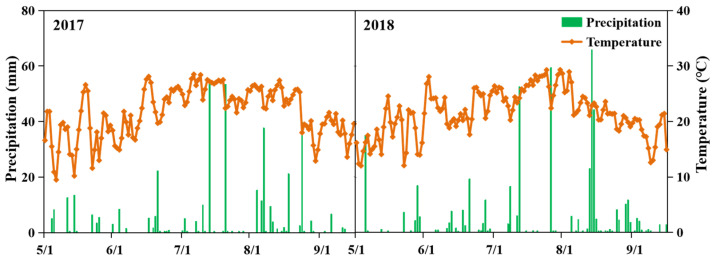
Temperature and precipitation during the test.

A two-year (2017 and 2018) field experiment was conducted at the Lishu Experimental Station of the China Agricultural University (43.17° N, 124.26° E) on the Northeast China Plain. The mean annual temperature during the maize growth season (from May to September) was 21.0 °C in 2017 and 20.7 °C in 2018, and the average annual precipitation was 474.7 mm in 2017 and 471.0 mm in 2018, respectively (Figure 6). The soil properties in the top 20 cm were as follows: bulk density 1.52 g cm^−3^, pH 6.1, organic matter 10.4 g kg^−1^, total nitrogen (N) 1.2 g kg^−1^, available phosphorus (Olsen-P) 35 mg kg^−1^, available potassium (NH_4_OAc-K) 157 mg kg^−1^ and mineral N (NO_3_^−^-N and NH_4_^+^-N) 24.5 mg kg^−1^. 

### 4.2. Field Experiments and Crop Managements

The experimental design consisted of a completely randomized block with three replicates, with an area of 5 m × 8 m for each plot. A high-yielding maize hybrid (Liang Yu 99) was used in this study. The planting density was 65,000 plants ha^−1^. All N fertilizer was applied in furrows to the top 10–15 cm of the soil.

All N treatments were applied at a total rate of 180 kg N ha^−1^ (UAN, N, 32%) except the control treatment without N. Each plot was supplied with 90 kg P_2_O_5_ ha^−1^ (Ca(H_2_PO_4_)_2_, P_2_O_5_ 46%) and 90 kg K_2_O ha^−1^ (KCl, K_2_O 60%) at sowing with basal N fertilizer. 

Three inhibitors were employed in the trial: urease inhibitor NBPT, nitrification inhibitor DCD and DMPP. A single or a combination of them will be added to UAN, the seven treatments were: (i) without N (N0), (ii) UAN, (iii) UAN + NBPT, (iv) UAN + DMPP, (v) UAN + DCD, (vi) UAN + NBPT + DCD and (vii) UAN + NBPT + DMPP.

### 4.3. Grain Yield

Maize was harvested on 4 October in two years, and 18 m^2^ in the middle of each plot was used for yield determination. All fresh ears were shelled and weighed in the field. Some of the fresh grains were oven-dried to determine the grain moisture content. Grain yield was expressed at 14.0% moisture.

### 4.4. N_2_O and NH_3_ Emissions Measurement

N_2_O emission was measured manually using the closed static chamber method as described by Zheng et al. (2008) [45]. Each chamber was composed of a 60 cm × 60 cm × 60 cm top chamber and a stainless steel base frame (60 cm × 30 cm × 15 cm). To avoid the sharp increase in air temperature inside the chamber in summer during the sampling period, each side of the top chamber was covered with Styrofoam coating. Additionally, two small fans were installed at opposite positions at the top of each chamber to ensure complete mixing of air inside the chamber. The gas sampling was performed from 8:00 to 10:00 h to best represent the mean daily flux. After the fertilization and precipitation events, gas samples were collected at an interval of 1–2 days for about 10 and 5 days, respectively, depending on when the gas fluxes decreased to the normal level, ultimately totaling 70 gas samplings every year, using 60 mL plastic syringes through a three-way stopcock and a Teflon tube connected to the chamber at 0, 10, 20 and 30 min after the chambers were closed. 

The N_2_O concentration was analyzed on the sampling day using gas chromatography (Agilent 7890A, Shanghai, China) equipped with an electronic capture detector (ECD). High-purity di-nitrogen (N_2_) (99.999%) was used as carrier gas at a flow rate of 21 mL min^−1^. The ECD detector and column oven temperatures were 300 and 60 °C, respectively. The N_2_O fluxes was calculated from the linear increase in the concentrations in the chamber during the sampling period, and the cumulative emissions were estimated using linear interpolation.

**Figure 7:**
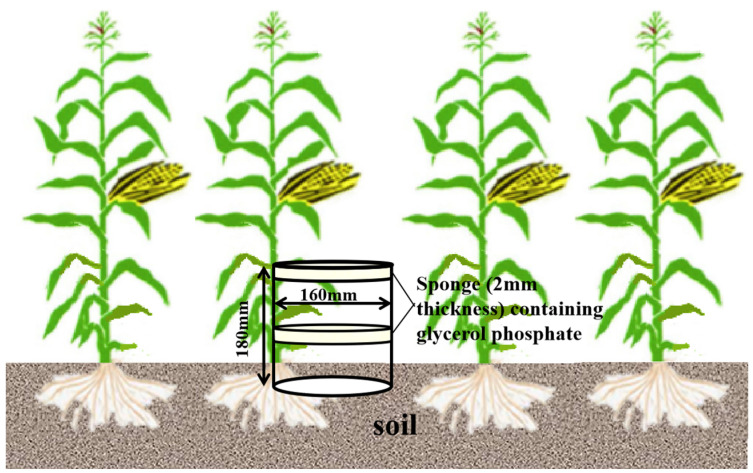
Schematic diagram of NH_3_ emission monitoring.

NH_3_ emission was measured by continuous monitoring for 7–10 days after fertilization. To detect NH_3_ emission, a method similar to the semi-open sponge method. A general schematic of the apparatus was shown in Figure 7. Three units were placed in each plot. Two sheets of filter paper (130 mm diameter and 2 mm thickness) containing oxalic acid/glycerol were placed inside each cylinder on a wire rack. A gasket made of a strip of polyfoam provided a tight fit between the sponge and the PVC tube. The upper sponge served to prevent the contamination of the inner system with atmospheric ammonia, whereas the lower sponge took up the ammonia volatilized from the soil. After fixed periods of exposure, the sponges were removed from the tube on the sampling dates. The NH_4_^+^ in each sponge was extracted by 1 M KCl solution, and the extracts were analyzed using an automated flow injection analyzer (FLOWSYS, Italy).

### 4.5. Plant and Soil Sampling and Analysis

Three representative plants in each plot were sampled close to the ground and then separated into three parts: leaf, stem and grain. All samples were first oven-dried at 105 °C for 30 min and then to constant weight at 70 °C. All dried samples were ground to make powder (<0.15 mm) with a ball mill for total N content analysis (Wolf, 1982).

N recovery efficiency (NRE) was calculated as follows:


NRE (%) = (N uptake in fertilized treatment − N uptake in unfertilized treatment)/N rate × 100
(1)

Mineral N (NO_3_^−^-N and NH_4_^+^-N) in the soil profile was monitored by sampling two replicate soil cores at five soil depths (0–20, 20–40, 40–60, 60–80 and 80–100 cm) with an auger before sowing and after harvest. Representative subsamples were extracted by shaking with 0.01 M CaCl_2_ (soil: solution ratio 1:10) for 1 h on a rotary shaker (180 rev min^−1^) and followed by filtration. The extracts were analyzed by an automated continuous flow analyzer (AA_3_, Germany).


Apparent N residual rate (%) = (Residual N in fertilized treatment − Residual N in unfertilized treatment)/N rate × 100
(2)

Apparent N loss rate (%) = 100 − N recovery efficiency − Apparent N residual rate
(3)

### 4.6. Statistical Analyses

All data across the years and treatments were analyzed using analysis of variance (ANOVA) with a two-factor analysis program by using the SAS statistical package (SAS Institute Inc., Cary, NC, USA). The least significant difference (LSD) test was used to determine the significance of difference among treatments at the 0.05 probability level. All figures were made using Origin 8.5 software (Origin Lab Corporation, Northampton, MA, USA).

## 5. Conclusions

In our study, the addition of UI, NI and DI to UAN effectively improved the agronomic and environmental effects of maize. DCD and DI treatments (NBPT + DCD, NBPT + DMPP) significantly increased corn yield by 8.1–21.2% compared with UAN, but the yield increase rate of DCD was 13.1 percentage points higher than that of NBPT + DCD, which means that combination of NBPT and DCD in UAN could not play a better role. UI and DI reduced NH_3_ emissions by 3.5–13.0% compared with UAN, while NI had the opposite effect; NI and DI reduce N_2_O emissions by 3.8–12.0% compared with UAN, and UI had the opposite effect, which indicated that the addition of DI to UAN is an effective way to reduce gas emissions. In general, adding different kinds of inhibitors in UAN can significantly reduce nitrogen loss by 7.0–28.1%; NI and DI can significantly improve nitrogen utilization efficiency by 24.5% and 30.27% and reduce nitrogen residue by 18.7% and 7.8%, respectively, and from the various combinations, NBPT + DMPP has the best effect. Therefore, it is recommended to add the combination of NBPT and DMPP to obtain the best agronomic and environmental effects when applying urea–ammonium nitrate solution on maize.

## Figures and Tables

**Table 1:** Total NH_3_ emission and N_2_O emission of different treatments in 2017 and 2018.

Treatments	NH_3_ Emission		NH_3_ Emission Factor		N_2_O Emission		N_2_O Emission Factor	
	(kg N ha^−1^)		(%)		(kg N ha^−1^)		(%)	
	2017	2018	2017	2018	2017	2018	2017	2018
N0	5.2 ± 0.6 Be	6.1 ± 1.0 Ae			0.09 ± 0.02 Bf	0.18 ± 0.04 Af		
UAN	18.3 ± 1.2 Bc	21.6 ± 3.2 Abc	7.3 ± 1.0 Bc	8.6 ± 1.6 Abc	1.72 ± 0.27 Bb	1.94 ± 0.19 Ab	0.91 ± 0.20 Bb	0.98 ± 0.22 Ab
NBPT	16.2 ± 1.1 Bd	18.5 ± 2.8 Ad	6.1 ± 0.9 Bd	6.9 ± 1.3 Ad	1.91 ± 0.30 Ba	2.08 ± 0.24 Aa	1.01 ± 0.25 Aa	1.06 ± 0.21 Aa
DMPP	20.3 ± 1.1 Bb	23.2 ± 3.1 Ab	8.4 ± 1.1 Bb	9.5 ± 1.8 Ab	1.51 ± 0.18 Be	1.71 ± 0.13 Ae	0.79 ± 0.19 Bd	0.85 ± 0.14 Ad
DCD	22.6 ± 1.3 Ba	27.8 ± 2.2 Aa	9.7 ± 1.2 Ba	12.0 ± 2.2 Aa	1.55 ± 0.13 Bde	1.72 ± 0.11 Ae	0.81 ± 0.18 Bd	0.86 ± 0.12 Ad
NBPT + DCD	17.9 ± 1.1 Bc	21.4 ± 1.4 Abc	7.1 ± 1.0 Bc	8.5 ± 1.9 Abc	1.66 ± 0.22 Bc	1.86 ± 0.16 Ac	0.87 ± 0.19 Bc	0.94 ± 0.16 Ac
NBPT + DMPP	17.0 ± 1.0 Bc	19.9 ± 1.9 Acd	6.6 ± 0.7 Ad	7.0 ± 1.2 Acd	1.59 ± 0.14 Bcd	1.80 ± 0.15 Ad	0.84 ± 0.16 Bcd	0.90 ± 0.14 Acd
ANOVA								
T	***		***		***		***	
Y	***		**		***		**	
T×Y	***		**		***		**	

Note: N0, without N; UAN, Urea–ammonium nitrate; NBPT, N-(n-buty_l_) thiophosphoric triamide; DMPP, 3,4-dimethylpyrazole phosphate; DCD, dicyandiamide. ** and *** mean significant at *p* < 0.01 and *p* < 0.001, respectively. Different capital letters above the bars indicate significant differences among years at *p* < 0.05. Different lowercase letters above the bars indicate significant differences among treatments at *p* < 0.05. Values in columns marked with at least the same letter do not differ significantly (*p* = 0.05).

**Table 2:** Nitrogen budgets (kg ha^−1^) in 2017 and 2018.

Year	Item	N0	UAN	NBPT	DMPP	DCD	NBPT + DCD	NBPT + DMPP
2017	A. N input							
	1. Spring inorganic N ^a^	93	93	93	93	93	93	93
	2. Applied N	0	180	180	180	180	180	180
	3. Apparent N mineralization ^b^	70	70	70	70	70	70	70
	Total input: 1 + 2 + 3	163	343	343	343	343	343	343
	B. N output							
	4. Plant N uptake	122	179	183	189	202	192	203
	5. Residual N ^c^	41	67	71	62	60	65	66
	6. NH_3_ and N_2_O emissions	5.3	20	18	22	24	20	19
	7. Apparent N losses ^d^	0	77	71	70	57	66	55
	8. N surplus ^e^	41	87	89	84	84	85	85
2018	A. N input							
	1. Spring inorganic N ^a^	41	67	71	62	60	65	66
	2. Applied N	0	180	180	180	180	180	180
	3. Apparent N mineralization ^b^	92	92	92	92	92	92	92
	Total input: 1 + 2 + 3	133	339	343	334	332	337	338
	B. N output							
	4. Plant N uptake	110	184	186	196	204	196	208
	5. Residual N ^c^	23	63	68	58	55	57	62
	6. NH_3_ and N_2_O emissions	6.3	24	21	25	30	23	22
	7. Apparent N losses ^d^	0	68	68	55	43	61	47
	8. N surplus ^e^	23	87	89	83	85	80	83

Note: N0, without N; UAN, Urea–ammonium nitrate; NBPT, N-(n-buty_l_) thiophosphoric triamide; DMPP, 3,4-dimethylpyrazole phosphate; DCD, dicyandiamide; ^a.^ Samples collected on 15 April 2017 and 9 April 2018; ^b.^ Apparent N mineralization = N uptake (N0) + N residual (N0)—Spring inorganic N (N0). ^c.^ Samples collected on 20 December 2017 and 22 December 2018; ^d.^ Apparent N loss = (spring inorganic N + applied N + apparent N mineralization) − (plant N uptake + residual N + NH_3_ and N_2_O emissions); ^e.^ N surplus = (residual N + apparent N losses).

**Table 3:** The fate of UAN with different inhibitors in both maize growing seasons.

Treatments	NRE (%)		N Residual (%)		N Losses (%)	
	2017	2018	2017	2018	2017	2018
UAN	31.6 ± 1.8 Bd	41.4 ± 1.9 Ac	14.4 ± 1.9 Bab	22.2 ± 1.6 Ab	54.0 ± 3.2 Aa	36.4 ± 1.8 Ba
NBPT	33.7 ± 1.2 Bd	42.4 ± 3.2 Ac	16.4 ± 1.6 Ba	25.0 ± 1.8 Aa	49.9 ± 2.7 Ab	32.6 ± 2.5 Bb
DMPP	37.1 ± 2.3 Bbc	47.8 ± 3.3 Ab	11.7 ± 0.9 Bc	19.3 ± 2.1 Abc	51.2 ± 2.9 Ab	32.9 ± 2.4 Bb
DCD	44.7 ± 2.9 ABa	52.1 ± 2.9 Aa	10.6 ± 1.1 Bc	17.9 ± 1.5 Ad	44.7 ± 1.9 Ac	30.0 ± 1.7 Bc
NBPT + DCD	40.6 ± 1.2 Bb	50.0 ± 1.0 Aab	13.3 ± 1.4 ABb	18.8 ± 1.7 Ac	46.1 ± 2.1 Ac	31.2 ± 1.3 Bc
NBPT + DMPP	45.3 ± 1.2 Ba	54.3 ± 3.6 Aa	14.0 ± 1.6 Bb	21.4 ± 2.0 Ab	40.7 ± 1.4 Ad	24.3 ± 1.1 Bd
ANOVA						
T	***		***		***	
Y	**		**		***	
T×Y	**		**		***	

Note: UAN, Urea–ammonium nitrate; NBPT, N-(n-buty_l_) thiophosphoric triamide; DMPP, 3,4-dimethylpyrazole phosphate; DCD, dicyandiamide. ** and ***, significant at *p* < 0.01 and *p* < 0.001, respectively. Different capital letters above the bars indicate significant differences among years at *p* < 0.05. Different lowercase letters above the bars indicate significant differences among treatments at *p* < 0.05. Values in columns marked with at least the same letter do not differ significantly (*p* = 0.05).

## Data Availability

The data are available upon request.
